# Association of Parental Screen Addiction with Young Children’s Screen Addiction: A Chain-Mediating Model

**DOI:** 10.3390/ijerph191912788

**Published:** 2022-10-06

**Authors:** Hui Li, Wenwei Luo, Huihua He

**Affiliations:** Shanghai Institute of Early Childhood Education, Shanghai Normal University, Shanghai 200234, China

**Keywords:** parental screen addiction, children’s screen addiction, parental anxiety, parent–child relationship

## Abstract

Preschool children are immersed in screen media, yet little study has been conducted on screen addiction among them. This study aimed to investigate the relationship between parental screen addiction and young children’s screen addiction and to verify factors that mediate this relationship. A total of 477 parents of kindergarteners (3–6 years old) were recruited via six kindergartens in Henan province, China. They completed the Problematic Media Use Measure Short Form of Children, the Self-Rating Anxiety Scale, the Child–Parent Relationship Scale, and the Parental Screen Addiction Scale. The results showed that the relationships between each pair of parental screen addiction, parental anxiety, and children’s screen addiction were significantly positive, but the parent–child relationship was negatively correlated with the other variables. Parental screen addiction can directly and indirectly affect children’s screen addiction through parental anxiety and the parent–child relationship. The findings contribute to the development of family protection mechanisms against screen addiction in children.

## 1. Introduction

Children are currently living in an age of screen-based media culture where a wide variety of screen terminals are profoundly influencing the way they live [1,2]. Previous studies indicated that toddlers and preschoolers have increasing screen ownership and time spent [3,4,5,6,7]. For instance, multiple studies have shown that children in countries, such as China, India, and the United States, have screen exposure times that exceed the limits recommended by the American Academy of Pediatrics (<1 or <2 h/day) [7,8,9]. Meanwhile, researchers argued that excessive screen media use can lead to physical and psychological problems in children, such as obesity, myopia, sleep problems, and anxiety [10,11,12]. Thus, the negative impact of screen time on children’s physical and mental health is a public health issue of widespread international concern [13].

However, screen time was confirmed significantly positively correlated with screen addiction, and excessive screen exposure time may increase the incidence of screen addiction [8,14,15]. A large body of research has focused on exploring screen exposure time in preschool children [3,7,16], while little research has been conducted on screen addiction. Nevertheless, the excessive screen exposure time should not be equated with problematic screen media use [17]. Furthermore, screen addiction in adolescents is of wide interest, but little is known about screen addiction in young children [8,18]. Hence, it is very urgent to examine the current status and mechanism of screen addiction in preschool children. While parental screen addition could lead to young children’s screen addiction, the mechanisms of how parental screen addiction affects children’s screen addiction are not yet clear [14]. Therefore, this study aims to explore the factors that predict young children’s screen addiction and the mechanism underlying it to lay a solid foundation for developing strategies to protect young children from screen addiction which will contribute to the existing children’s screen addiction literature.

### 1.1. Parental Screen Addiction (PSA) and Children’s Screen Addiction (CSA)

Screen addiction (e.g., TV, smartphone, computer, tablet, etc.) can be defined as excessive, uncontrolled, and obsessive media consumption using screen media devices [15,19]. It is the psychological and behavioral withdrawal symptoms of individuals when they are disconnected from the context of using screen media such as smartphones, tablets, TV, etc. [19]. Thus, Internet addiction, digital game addiction, computer addiction, TV addiction, smartphone addiction, etc., all belong to the subtypes of screen addiction [1,18,20]. Based on the above research, this study considers children’s screen addiction (CSA) and parental screen addiction (PSA) as problematic screen media use, which is characterized by a continuum of unregulated and compulsive use of multiple types of screen devices. Lin et al. explained that screen addiction can manifest itself in maladaptive symptoms such as the inability to control cravings, withdrawal/avoidance, and loss of productivity [15]. Domoff etc. stated that addictive use in children may interfere with home life, school functioning, and social functioning [18]. However, children are incapable of being aware of the effects of screen addiction on these functions. In addition, the Interactional Theory of Childhood Problematic Media Use (IT-CPU) proposes parent media use and beliefs as proximal factors that likely contribute to children’s problematic screen use [21]. Therefore, although screen addiction in children is an individual problem, the family, as the most important environmental factor, must be taken into account [22].

Recent empirical studies have suggested that parental problematic media use could be an important predictor of children’s smartphone and Internet addiction [14,23,24,25,26,27]. For example, Lauricella et al. found that the amount of time children aged 0–8 spent watching the TV and on the computer, smartphone, and tablet was highly correlated with their parents’ own screen time [28]. Kim et al. revealed that maternal smartphone dependency significantly predicts adolescent smartphone dependency [29]. These results could be explained by an intergenerational transmission model indicating that children’s psychological problems can be intergenerationally transmitted [30]. In addition, combined with Social Learning Theory [31], children will observe and imitate their parents’ problematic screen media use, leading to the intergenerational transmission of screen addiction. Therefore, when parents are addicted to screen use, young children are at high risk of screen use addiction. Based on previous findings, we hypothesized that:

**H1.**  *Parents’ screen addiction can have an impact on their young children’s screen addiction.*

### 1.2. Parental Anxiety (PA) as a Mediator

Parental anxiety (PA) is likely to mediate between parental screen addiction and children’s screen addiction. Parental anxiety is a generalized anxiety disorder, which can be defined as neurosis characterized by excessive anxiety causing panic and is often accompanied by somatic symptoms [32]. Previous research examined the correlation between anxiety and digital media addiction [20,33,34,35,36]. Results of a meta-analysis study indicated that anxiety in adults and adolescents was positively associated with mobile phone addiction [37]. Chen et al. discovered that excessive screen time would negatively affect sleep quality and physical activity, which in turn would lead to higher levels of anxiety [34]. Moreover, according to the embodied Motivation Cognitive Theory, pleasant stimuli activate the appetitive system, while unpleasant stimuli activate the aversive system [38]. A previous experimental study demonstrated that when participants with mobile phone addiction were separated from their mobile phones, their measured physiological anxiety ratings increased significantly [39]. Therefore, when parents are separated from screen media such as mobile phones, aversive motivational systems are activated, which in turn can lead to anxiety [40]. Hence, screen addiction could be a significant predictor of anxiety [36,41,42,43], and we expect that parents with screen addiction are likely to have higher ratings of anxiety.

Moreover, previous research has demonstrated that psychological problems such as parental anxiety and depression are associated with externalizing problems in children [44]. As a result, children’s external behavioral problems such as Internet addiction, phone addiction, and tablet addiction are linked to negative emotional problems in their parents [44,45,46,47,48]. An empirical study based on a national sample in the USA showed that internalized psychological problems such as parental anxiety, depression, and withdrawal were strongly associated with their children’s time spent on screen activities such as watching television and videos and playing games [11]. This is probably because parents with mental health problems may have fewer psychological resources to monitor their child’s psychological and behavioral development, thus failing to provide positive alternatives to their child’s redundant screen media activities [11,45,49]. Hence, based on the theoretical and literature evidence stated above, we hypothesize that:

**H2.**  *PA plays a mediating role in the impact of PSA and CSA.*

### 1.3. Parent–Child Relationship (PCR) as a Mediator

Family Dynamics Theory indicates that family relationships are an important predictive factor of children’s media addiction [25,50,51]. Past studies have found that the quality of the parent–child relationship (PCR) was significantly negatively associated with mobile phones and Internet addiction among children [51,52,53,54]. A systematic review study also revealed that parent–child attachment was a negative predictive factor of child problematic touch screen device use [13]. According to the Expectancy Violations Theory, children who do not achieve psychological satisfaction from parent–child interactions can have increased feelings of isolation and insecurity, which in turn may exacerbate the risk of problematic media use [27,46,55].

In addition, The Displacement Hypothesis Theory suggests that excessive use of social media will replace real-world social interaction and reduce social engagement and relationship satisfaction [24]. Based on this theory, parental excessive screen time may substitute meaningful parent–child interactions, thus negatively impacting the parent–child relationship [26,56,57]. Past studies have found that parents’ phubbing behavior can make children feel neglected, which can weaken parent–child attachments [27,58]. Given the evidence above, we hypothesized that:

**H3.**  *PCR plays a mediating role in the association between PSA and CSA.*

### 1.4. The Chain Mediation Effect of Parental Anxiety and Parent–Child Relationship

**Figure 1:**
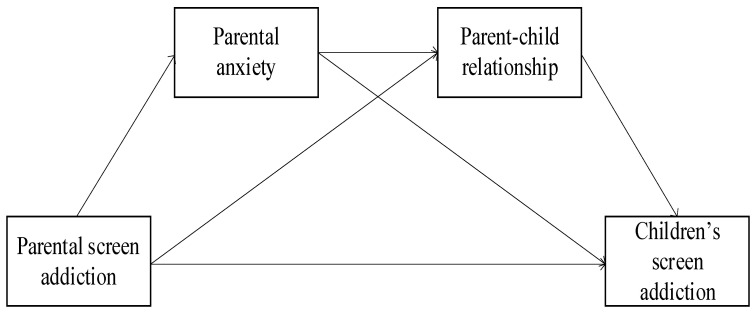
Hypothesized mediation model.

Offline face-to-face communication is an important factor in the development of interpersonal relationships. However, a previous study indicated anxiety was negatively related to willingness to communicate face-to-face [33]. Thus, parental anxiety may reduce the desire for parent–child communication, thereby reducing parent–child relationship closeness. In addition, according to Emotional Security Theory, parents’ emotional problems can affect children’s perception of the parent–child relationship, which leads to the emergence of children’s externalizing problems [46]. Meanwhile, the Spillover Hypothesis Theory states that emotions or behaviors within a family system are transferred from one relationship to another with the same valence [59]. Therefore, parental anxiety may spill over into the parent–child relationship, negatively affecting the developmental adaptation of children and adolescents through negative parent–child interactions [60]. Therefore, the parent–child relationship could be affected by parental anxiety. Meanwhile, children may use the screen medium to compensate for their dissatisfaction with poor parent–child interaction [61,62]. Based on these, we hypothesized that:

**H4.**  *PA and PCR play a chain-mediating role in the relationship between PSA on CSA (Figure 1).*

## 2. Methods

### 2.1. Participants

A total of 477 parents of kindergarteners (3–6 years old) from Henan Province, China, participated in the current study. After obtaining permission from six kindergartens from five regions in Henan province, including Zhengzhou, Luoyang, Pingdingshan, Zhumadian, and Xuchang, questionnaires were distributed by WeChat, which is a popular social media APP in Mainland China. The study was reviewed and approved by the Ethics Committee of Shanghai Normal University. All participants signed informed consent prior to filling out the questionnaire. Among the participants, 387 were mothers (81.1%), and 90 were fathers (18.9%); 257 were parents of boys (53.9%), and 220 were parents of girls (46.1%); 109 were 3–4 years old (22.9%), 124 were 4–5 years old (26.0%), and 244 were 5–6 years old (51.1%).

### 2.2. Measures

#### 2.2.1. Children’s Screen Addiction

Screen addiction in young children was evaluated by The Problematic Media Use Measure Short Form (PMUM–SF). The PMUM–SF was developed by Domoff et al. to assess screen media addiction in children ages 4–11 years old [18]. The scale includes nine items and should be completed by the parent of the child. The nine items correspond to the nine dimensions of unsuccessful control, loss of interest, preoccupation, psychosocial consequences, serious problems due to use, withdrawal, tolerance, deception, and escape/relieve mood. Items are scored on a 5-point Likert scale from 1 (never) to 5 (always). Higher scores indicate higher levels of screen addiction. The Cronbach alpha coefficient of PMUM–SF in this study was 0.91.

#### 2.2.2. Parental Screen Addiction

To evaluate parental screen addiction,10 items were adapted from Xiang’s 10-item short version of the smartphone addiction scale (SAS-SV), which was derived from Kwon [63,64]. Without any established measure, we adapted SAS-SV items by replacing “Smartphone” with “Screen devices such as smartphones”. Sample items included the following: “Missing planned work due to screen devices use such as smartphone”, “Will not be able to stand not having a screen device such as a smartphone”, and “The people around me tell me that I use my screen devices such as a smartphone too much.” All items use a 6-point Likert scale from 1 (strongly disagree) to 6 (strongly agree). Higher scores indicate stronger screen addiction. If children’s father and mother scored more than 31 and 33, respectively, they were considered as screen addicts [63]. Cronbach’s α of the scale was 0.90 in this study.

### 2.3. Parental Anxiety

The Self-Rating Anxiety Scale (SAS) compiled by Zung was used for measuring the level of Self-Rating Anxiety in this study [32]. The scale consists of 20 items rated on a 4-point Likert-type scale, ranging from “No/little time” (scored 1 point) to “Most/all of the time” (scored 4 points). The standard score is obtained when the total rough score is multiplied by 1.25. A higher score indicates a higher degree of anxiety. If participants’ score exceeds 50 points, they are considered as having anxiety symptoms. The Cronbach alpha coefficient of parental anxiety in this study was 0.80.

### 2.4. Parent–Child Relationship

The quality of the parent–child relationship was assessed by the short form of the Child–Parent Relationship Scale (CPRS-SF) [65]. Chinese scholars Zhang and Deng, respectively, conducted Chinese translation and revision of the CPRS-SF [66,67]. The scale consists of two dimensions: closeness (7 items) and conflict (8 items). It is a parental self-report instrument rating the items on a 5-point scale from 1 (completely disagree) to 5 (completely agree). The total score for the parent–child relationship is the sum of the closeness score and reversed conflict score in this study [68]. Higher scores indicate a better parent–child relationship. Many Chinese scholars have applied this scale in previous studies. For instance, Gong et al. examined the mediating role of the parent–child relationship on parental smartphone addiction and adolescent smartphone addiction with the CPRS-SF [14]. In this study, the Cronbach alpha coefficient for the whole scale was 0.85. The Cronbach alpha coefficients for closeness and conflict were 0.82 and 0.85, respectively.

### 2.5. Data Analysis

IBM SPSS 26.0 (IBM, Armonk, NY, USA) was used to preliminarily analyze the data. Harman’s single factor test was used to examine the common method bias for data validity. The descriptive data of PSA, CSA, PA, and PCR were reported using mean and standard deviation(M ± SD). Pearson correlation analysis was used to examine the correlation between each variable. To test the proposed mediating effect model, we used the PROCESS macro in SPSS (model 6). In addition, we generated 5000 bootstrap samples with retraction to estimate the 95% confidence interval of the mediating effect value.

## 3. Results

### 3.1. Common Method Bias

All measures in this study were self-reported scales filled in by parents, which may lead to common method bias. Hence, Harman’s single-factor method was employed, and results suggested 10 common factors with eigenvalues greater than one. The variance explained by the first of these factors was 22.63%, which was less than the critical value of 40%. It indicates that there was no significant common method bias in this study.

### 3.2. Descriptive Analysis and Correlation Analysis

The means, standard deviations, and correlation coefficient of variables in the mediation model were examined (Table 1). The means and standard deviations (M ± SD) of children’s screen addiction, parental screen addiction, parental anxiety, and parent–child relationship were 18.53 ± 6.34, 28.80 ± 10.16, 39.75 ± 8.32, and 61.25 ± 8.58, respectively.

In addition, the results of the correlation analysis indicated that children’s screen addiction was significantly positively correlated with parental anxiety (*r* = 0.37, *p* < 0.01) and parental screen addiction (*r* = 0.35, *p* < 0.01) but negatively correlated with parent–child relationship (*r* = −0.49, *p* < 0.01). Moreover, screen addiction of parents was significantly positively correlated with parental anxiety (*r* = 0.34, *p* < 0.01) but negatively correlated with parent–child relationship (*r* = −0.36, *p* < 0.01). Furthermore, a significantly negative correlation was found between parental anxiety and the parent–child relationship (*r* = −0.54, *p* < 0.01).

### 3.3. Relationship between PSA and CSA: A Chain–Mediating Effect Analysis

The above analysis showed that there was a significant correlation between the variables and that collinearity may exist. Therefore, before testing the effect, the predictive variables in the equation were standardized, and collinearity diagnostics were performed. The results showed that the variance inflation factors (1.19, 1.46, and 1.48) of all of the predictors were less than five. Therefore, there was no serious collinearity in the data used for this study, indicating that they were suitable for further mediation effect tests. Then, model 6 from the PROCESS was used to examine the 95% confidence interval (CI) of the mediating effects of PA and PCR on the impact of PSA and CSA (the bootstrap sample size was 5000).

The results showed that parental screen addiction positively predicted children’s screen addiction (*β* = 0.35 *p* < 0.001). When parental anxiety and the parent–child relationship were added as mediators, PSA still positively predicted CSA (*β* = 0.19, *p* < 0.001). Meanwhile, parental screen addiction positively predicted parental anxiety (*β* = 0.34, *p* < 0.001) but negatively predicted the parent–child relationship (β = −0.20, *p* < 0.001). Parental anxiety positively predicted CSA (*β* = 0.11, *p* < 0.05) but negatively predicted the parent–child relationship (*β* = −0.47, *p* < 0.001). Meanwhile, the parent–child relationship negatively predicted CSA (*β* = −0.36, *p* < 0.001).

The results of the mediation effect analysis suggested that parental anxiety and the parent–child relationship play an incomplete mediating role between PSA and CSA. This means that both the direct and indirect effects of parental screen addiction on children’s screen addiction are significant. As is shown in Table 2, the size of the mediation effect was 0.17, 95% CI = [0.12, 0.23], *SE* = 0.03, and the ratio of the mediating effect to the total effect was 47.46%.

**Figure 2:**
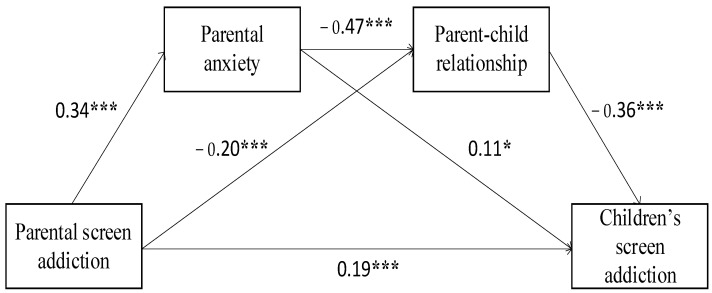
Standardized path coefficients for serial multiple mediations. Note. *** *p* <0.001, * *p* <0.05.

Specifically, the mediating effect consists of indirect effects arising from three pathways. The size of indirect effect one via the PSA→PA→CSA pathway was 0.40, 95% CI = [0.01, .08], *SE* = 0.02; the size of indirect effect two via the PSA→PA→PCR→CSA pathway was 0.06, 95% CI = [0.04, 0.09], *SE* = 0.01; The size of indirect effect three via the PSA→PCR→CSA pathway was 0.07, 95% CI = [0.04, 0.11], *SE* = 0.02; The contribution rates of the three indirect effects in the total effect were 11.02%, 16.38%, and 20.06%, respectively. The indirect effects were significant based on bootstrap 95% confidence intervals. The detailed path model is shown in Figure 2. Therefore, Hypotheses 1, 2, 3, and 4 are supported.

## 4. Discussion

The current study used a sample of pre-school children in Henan, China, to verify the relationship between PSA and CSA via a chain mediation model. Results showed that PSA significantly positively predicted CSA, indicating that children had a higher tendency toward screen addiction when their parents’ showed problematic screen media use. In addition, the findings confirm that parental anxiety and parent–child relationship mediate the relationship between parental screen addiction and children’s screen addiction, respectively. Meanwhile, the results also suggest that parental anxiety and the parent–child relationship had a significant chain mediation effect on PSA and CSA. Therefore, all hypotheses were supported, and the chain mediating mechanism was developed by which parental screen addiction affects preschool children’s screen addiction.

### 4.1. The Relationship between PSA and CSA

The present study confirmed that parental screen addiction is a risk factor for children’s screen addiction, which is support for the intergenerational transmission model and the Social Learning Theory [30,31]. As significant others to children, parents’ perceptions and behaviors become objects for children to observe and imitate. Therefore, excessive use of screen media by parents can create poor role models for children to learn from, thus increasing the likelihood of screen addiction in children. In addition, as with previous findings on adolescent populations, screen addiction in preschoolers can be influenced by parental problematic screen media use with regard to smartphones and Internet use [14,23,24,26]. By comparison, pre-school children, with their immature minds and poor self-control, are more susceptible to the negative influences of their home environment. Parents of pre-school children should be more concerned about the effect of their own screen media use on their children. Therefore, parents should regulate their own use of screen media, for example, by not using screen media excessively in their daily lives to set a good learning example for their children.

### 4.2. The Mediating Role of Parental Anxiety

This study also found that parental anxiety (PA) mediated the relationship between PSA and CSA. That is, the more parents are addicted to screen media use, the greater the anxiety generated by the individual, which increases the risk of screen addiction in children. Hence, in order to reduce the negative impact of parental anxiety on children’s screen addiction, it is important for parents to maintain their own mental health by adopting good daily habits and positive social interactions. The result of PSA leading to an increment in parental anxiety is consistent with the findings of Elhai et al. [42]. Parents who significantly overuse screen media can have reduced social competence and sense of security, which can lead to negative emotions such as anxiety and depression. In addition, excessive use of screen media may also affect the activity of the pineal gland in the brain, reducing the quality of sleep and leading to negative feelings of anxiety [34,37].

Meanwhile, the result of parental anxiety leading to an increase in children’s screen addiction is different from the findings of Lam [48]. This may be because the subjects in the two studies were different, preschoolers and adolescents, respectively. This may indicate that child age plays an important moderating role in the relationship between parental anxiety and child addiction. In conclusion, parental screen addiction and parental anxiety as externalizing and internalizing psychological problems are closely related to problematic screen media use by children [11]. To our knowledge, few studies have explored models of the mediating role of parental anxiety in the relationship between PSA and CSA, so the present study was able to fill this gap.

### 4.3. The Mediating Role of the Parent–Child Relationship

We also identified that the parent–child relationship (PCR) plays a negative and mediating role in the relationship between PSA and CSA. Parental screen addiction negatively predicted the parent–child relationship, and the parent–child relationship negatively predicted children’s screen addiction. Some previous studies have shown that poor parent–child relationships can lead to psychological and behavioral problems in children, including addiction to media such as smartphones and the Internet, etc. [27,51,54]. Parents who are addicted to screen-based media such as smartphones may be less sensitive to their children’s needs and parent–child attachments [13]. Meanwhile, Bernardi and Pallanti demonstrated that poor family relationships may lead to increased child use of Internet-connected devices as a way to escape conflict and emotional distress [69]. The parent–child relationship is an important part of the family relationship and a key factor in triggering media addiction in adolescents and children [14]. Therefore, improving the quality of parent–child relationships is crucial to reducing excessive media use by children [13]. In family parenting, parents need to be active companions to their young children and replace negative, excessive screen activities with positive ones, such as frequent parent–child reading, parent–child games and outdoor trips with their children.

### 4.4. The Chain-Mediating Role of Parental Anxiety and Parent–Child Relationship

We further found a serial mediation role for parental anxiety and the parent–child relationship in the relationship between PSA and CSA. Excessive, uncontrolled parental use of screen media can affect their generalized anxiety, which in turn can reduce the closeness of the parent–child relationship, thereby increasing the risk of screen addiction in children. To our knowledge, few studies have explored this model. Therefore, the current study advances our understanding of the mechanisms that link parental screen addiction and child screen addiction. Surprisingly, there was a significant correlation between parental anxiety and parent–child relationship in the present chain mediation model, which is inconsistent with the results of Russell’s study [70]. This may be due to the wide age range of the sample in the latter study, which included children aged 0–18 years. This also further suggests that the age of the child may play a key moderating role in parental anxiety and the parent–child relationships. A subsequent study also supports this view. MacNeill et al. stated that the interaction term between parental anxiety and child age significantly predicted parent-focused/controlling parenting attractor strength, but this significant correlation did not exist in children over 6.07 years old [71]. This may be because the younger children are, the more dependent they are on their parents, and the more they are influenced by their parents.

## 5. Conclusions

To sum up, screen addiction is an important challenge for children in the era of screen culture, and family factors are important predictors of screen addiction. This study verified the correlation between parental screen addiction, children’s screen addiction, parental anxiety, and parent–child relationship, and further proved the chain-mediated model of parental anxiety and the parent–child relationship between PSA and CSA. These findings suggest that to prevent children from becoming addicted to screen media, parents need to be good role models in the proper use of screen media, as well as maintain a good mental state and be active partners to children.

Despite the valuable results found in this study, there are still some notable limitations. First, the present study was based on a cross-sectional design, which may not reveal the true causal relationship. In the future, longitudinal cross-lagged studies or experimental designs are needed to confirm the causal relationship between PSA and CSA. Second, this study used parent-reported data for quantitative analysis and may not have examined the characteristics of the variables in sufficient depth. Thus, mixed research methods will be needed in the future to verify the relationships among the variables. Third, in addition to family factors, the children themselves may also be important process factors in the impact of parental problems on children’s externalizing problems [46]. However, the mediating model in this study did not include children’s individual factors (e.g., children’s anxiety/depression) as antecedent variables. Hence, the optimization process of the chain mediation model in this study needs to further consider the role of children’s individual factors. Fourth, the questionnaire used by this study relied on parental reports, but the ratio of fathers to mothers was not sufficiently balanced, which may have biased the results. Therefore, in the follow-up study, we need to conduct parent–child matching and ask both fathers and mothers to report on the questionnaire.

This study clarifies the mechanisms by which parental screen addiction affects children’s screen addiction. Moreover, we did not limit the study to a single type of screen device but extended it to a comprehensive range of screen device addictions. As a result, the findings have a wide range of application scenarios. Furthermore, the results of this study will provide parents with a better understanding of the impact of their factors on their child’s problematic use of screen media. They will also provide theoretical support for kindergartens to guide families in scientific childcare. In conclusion, this study can provide effective guidelines for family protection against screen addiction in young children and in turn promote the healthy development of children in the era of digital screen culture.

## Figures and Tables

**Table 1:** Descriptive analysis and inter-correlations among measures.

Variable	M	SD	1	2	3	4
1. Children’s screen addiction	18.53	6.34	-			
2. Parental screen addiction	28.80	10.16	0.35 **	-		
3. Parental anxiety	39.75	8.32	0.37 **	0.34 **	-	
4. Parent–child relationship	61.25	8.58	−0.49 **	−0.36 **	−0.54 **	-

Note. ** *p* < 0.01; average (M) using the total score.

**Table 2:** Standardized total, direct and indirect effects, and 95% confidence intervals.

Pathway	Estimate	*SE*	Ratio	95% Confidence Interval	
				Lower	Upper
PSA-PA-CSA	0.039	0.018	11.02%	0.008	0.078
PSA-PA-PCR-CSA	0.058	0.013	16.38%	0.037	0.088
PSA-PCR-CSA	0.071	0.018	20.06%	0.040	0.111
Total indirect effect	0.168	0.028	47.46%	0.118	0.226
Direct effect	0.186	0.042	52.54%	0.103	0.269
Total effect	0.354	0.043	100%	0.270	0.438

Note. PSA = parental screen addiction; CSA = children’s screen addiction; PA = parental anxiety; PCR = parent–child relationship.

## Data Availability

The data presented in this study are available on request from the corresponding author. The data are not publicly available due to ethical requirements.
